# Clinical Patterns and Factors Contributing to Ophthalmic and Otologic Events Associated With Immune Checkpoint Inhibitors: A Narrative Review

**DOI:** 10.7759/cureus.66611

**Published:** 2024-08-10

**Authors:** Amal M Sunyur, Duaa Alkhayat, Heba A Mohammad, Hussam A Alahmadi, Layan A Alharbi, Zakaria Y Khawaji, Ahmad S Badawi

**Affiliations:** 1 Medicine and Surgery, Taibah University, Medina, SAU; 2 Department of Clinical Oncology, Taibah University, Medina, SAU

**Keywords:** immunotherapy, hearing loss, non-auditory health effects, auditory effects, orbital effects, otologic events, ophthalmic events, immune checkpoint inhibitors

## Abstract

Immune checkpoint inhibitors, which are a type of cancer immunotherapy, have been associated with the development of adverse events related to an overactive immune system caused by the effect of this type of therapy. It affects a wide range of organs, including the ear and eye. Ophthalmic toxicity related to immune checkpoint inhibitors usually occurs bilaterally. Corneal toxicity (mainly dry eye disease) and uveitis are the most commonly reported patterns of toxicity. Other patterns of involvement include optic neuritis, serous retinal detachment, keratitis, ophthalmoplegia, and ocular myasthenia, but are not limited to them. Potential factors contributing to the development of toxicity are age, previous history of ocular immune disease, type, doses, and duration of treatment, and race. Ototoxicity is also reported in the literature, usually manifesting as bilateral, symmetrical/asymmetrical hearing loss. Ear toxicity presenting as ear fullness, tinnitus, and vertigo has also been mentioned in the literature. Hearing loss is often associated with word/speech recognition. An audiogram usually shows a pattern of sensorineural hearing loss. Otitis media has also been reported to be a potential cause of ear toxicity. Immune checkpoint inhibitor toxicity was present more commonly when used along with other anti-neoplastic agents. Ear toxicity, which presumably results from damage to the melanocytes in the ear, often presents with other melanocytotic manifestations, like uveitis and vitiligo. According to the literature, some agents (ipilimumab, nivolumab, atezolizumab, and pembrolizumab) were more commonly associated with toxic effects on the eye and ear and more when combined with each other.

## Introduction and background

**Figure 1:**
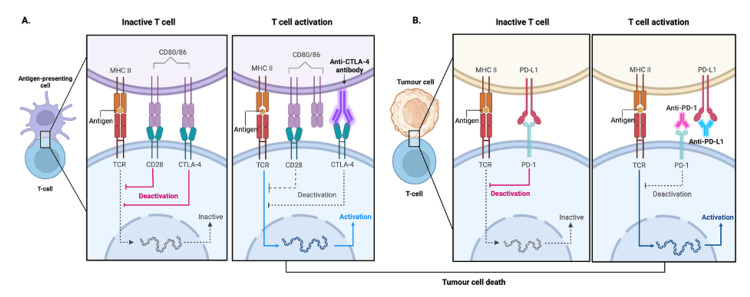
A visualization of the role of immune checkpoint proteins and the role of immune checkpoint inhibitors in enhancing T cell response. (A) Shows the mechanism by which cytotoxic T lymphocyte-associated protein-4 (CTLA-4), an immune checkpoint inhibitor, works. (B) Illustrates the function of the other two major checkpoint inhibitors, which are programmed cell death protein-1 (PD-1) and programmed death ligand-1 (PD-L1). Adapted from “Immune checkpoint proteins (CTLA-4, PD-1, and PD-L1) and their impact on T cell activity” by BioRender.com (2024). Retrieved from https://app.biorender.com/biorender-templates

Cancer is one of the most fatal conditions. Cancer treatments have advanced in the past few years through the use of immunotherapy. Immune checkpoints, which are molecules used to suppress chronic and/or aberrant immune activation, are used by malignant cells to prevent immune activation. Blocking immune checkpoint pathways using the monoclonal antibodies known as immune checkpoint inhibitors (ICIs), which are one of the ways to achieve anti-cancer immunity, reveals cancer cells to the immune system. Immune checkpoint molecules include cytotoxic T-lymphocyte associate protein-4 (CTLA-4), programmed death-1 (PD-1), and programmed death-ligand 1 (PD-L1) (Figure 1) [1,2].

Ipilimumab, which is the first FDA-approved ICI, and tremelimumab (CTLA-4 inhibiting agents) are usually prescribed for melanomas, renal cell carcinomas (RCC), non-small cell lung cancer (NSCLC), pleural mesotheliomas, and hepatocellular carcinomas (HCC). PD-L1 inhibiting agents (atezolizumab and durvalumab) are used mainly in small cell lung cancer (SCLC), NSCLC, HCC, melanomas (mainly atezolizumab), and urothelial cancer. PD-1 inhibitors (pembrolizumab and nivolumab), on the other hand, are the ICI class with the most diverse use. These can be used in skin cancer (both melanoma and non-melanoma cancers), HCC, RCC, NSCLC, SCLC, urothelial cancer, lymphomas, and head and neck malignancies [3].

**Figure 2:**
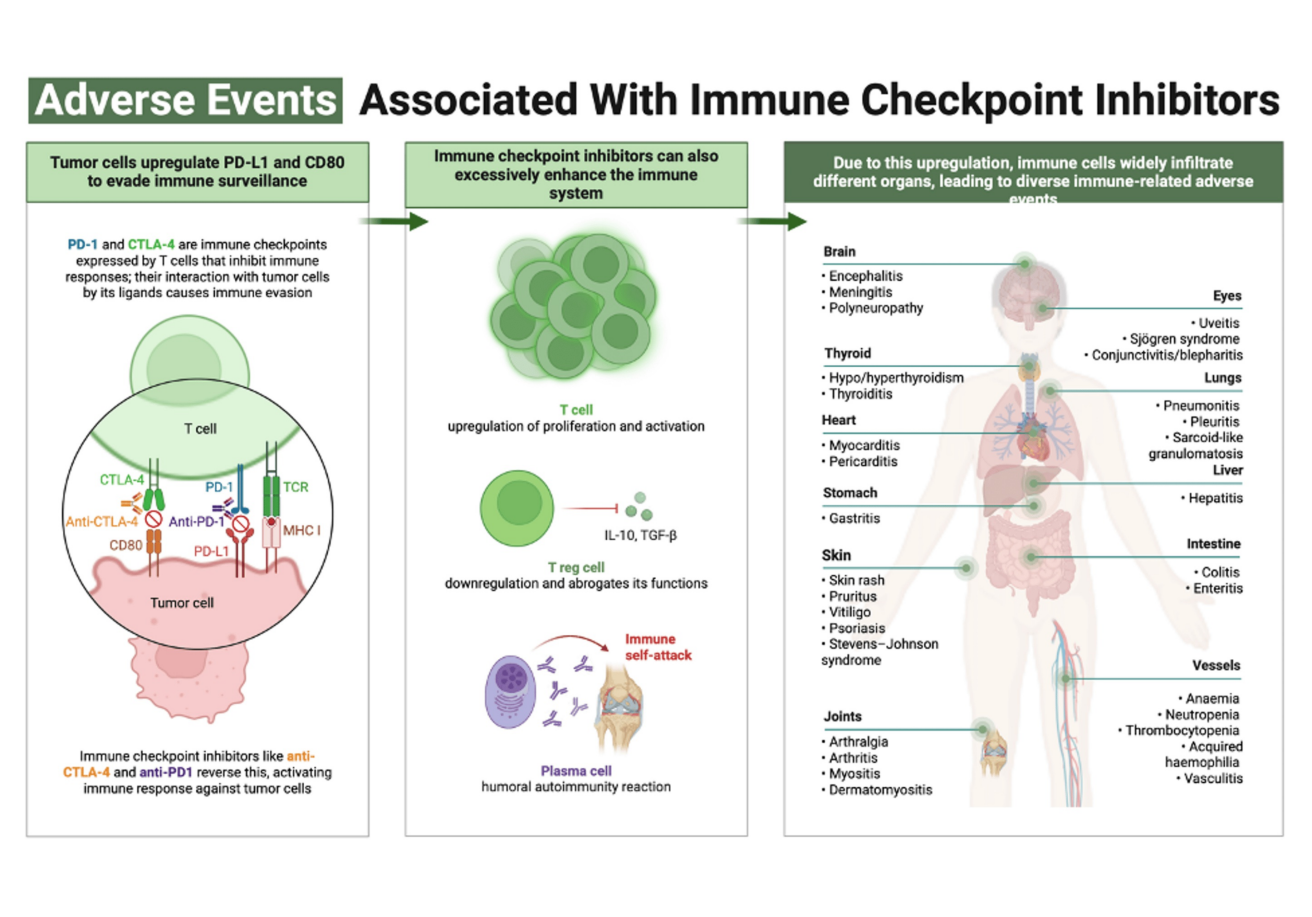
Different adverse events are associated with immune checkpoint inhibitors. Adapted from “Mechanisms of Immune-Related Adverse Events After Immune Checkpoint Inhibitors Immunotherapy” by BioRender.com (2024). Retrieved from https://app.biorender.com/biorender-templates

Cancer patients who are receiving an immune checkpoint-inhibiting agent may experience a set of adverse effects that occur in multiple organs (Figure 2). These events are often referred to as immune-related adverse effects (irAEs). Common events associated with ICIs include pneumonitis, colitis, dermatitis, arthritis, vasculitis, hepatitis, and endocrine dysfunction. Ocular events are commonly considered among the most commonly occurring events happening in relation to ICIs [4,5]. However, ototoxic events are often underrecognized [5].

Visual impairment has been reported in several studies to decrease the quality of life (QoL) of individuals. Multiple studies reported a decrease in quality of life in individuals with visual dysfunction, affecting the environmental, physical, and emotional domains of QoL [6]. Hearing dysfunction, additionally, was also reported to be associated with a lower QoL, impairing the ability of patients to carry out activities of daily living (financial management, transportation, shopping, etc.) and independent activities of daily living (toileting, walking, bathing, dressing, etc.), correlating with the severity of the dysfunction [7].

In this review, we aim to review the clinical features and factors associated with ICI-related ophthalmic and otologic toxicity, which may contribute to an impaired quality of life for patients, as the sensory functions related to these organs are vital in maintaining proper functioning. This is an attempt to recognize these irAE, and so further research on these areas and ways of rehabilitation can be done to improve the quality of life of individuals with otologic and visual impairment.

We searched for articles using Google Scholar and PubMed. The articles with better study designs (according to the hierarchy of evidence) were chosen for this review. Studies with topics that were investigated with a better study design were excluded. 

## Review

Ophthalmic events related to immune checkpoint inhibitors

Ophthalmic adverse events secondary to immune checkpoint inhibitors are rare, with low reported incidences in the existing literature. However, Abdel-Rahman (2017) conducted a meta-analysis that demonstrated the correlation between ophthalmic autoimmunity and the use of ICI. The analysis of the pool odds ratio of ocular toxicity was 3.40 (95% CI: 1.32-8.71; p = 0.01), denoting that ocular toxicity rates were considerably higher in patients who received ICI compared to non-ICI groups [8]. A survey-based study of 15 patients revealed that a subset of cases, predominantly those primarily treated with ipilimumab monotherapy or ipilimumab-nivolumab combination, developed autoimmune uveitis. The most common type was anterior uveitis, with bilaterality and mild-moderate severity seen in most documented cases. Other types of uveitis were reported, including posterior uveitis and panuveitis. Also, anterior chamber inflammation was observed in a few instances. These side effects, however, were transient, short in duration, and showed a good response to the standard corticosteroid treatment [9].

In a retrospective analytical study published in 2020, 231 patients received ipilimumab, while 88 were treated with ipilimumab+nivolumab and evaluated for eye-related adverse events. The findings indicated that among the entire cohort of patients, the ipilimumab group had the highest frequency of uveitis across its various types (anterior, posterior, intermediate, and panuveitis). These results underscore the increased likelihood of ocular complications associated with ipilimumab in contrast to other immune checkpoint inhibitors, particularly PD-1 inhibitors. Concerning the neuro-ophthalmic complications, the incidences of papilledema, optic atrophy, and cranial nerve (CN) three, four, and six palsies were as follows: 1492.5/100,000, 2238.8/100,000, and 746.3/100,000, respectively [10]. Additionally, orbital inflammation and ulcerative keratitis were reported in a case series, yet both were completely resolved with corticosteroids with no long-term visual sequelae [11].

A large-scale retrospective study involving over 1400 patients in a tertiary care center revealed only 15 cases of immune-related ocular side effects following the ipilimumab+nivolumumab regimen. Bilaterality was found in all patients except one. The toxicities that were seen among patients include myasthenia gravis, corneal punctate epithelial erosions, subconjunctival hemorrhage, corneal perforation, uveitis, hypotony maculopathy, cystoid macular edema, serous retinal detachment, choroiditis, Vogt-Koyanagi-Harada (VKH)-like syndrome, optic neuritis, and melanoma-associated retinopathy (MAR). Some cases exhibited chronicity, with findings of chronic uveitis, corneal scarring, persistent choroidal effusion, hypotony maculopathy, and bilateral optic neuropathy [12]. It’s worth noting that the new onset of visual symptoms among melanoma patients receiving immunotherapy may point toward a pathophysiological autoimmunity response. This was evident in three melanoma cases who complained of photopsia, night blindness, bilateral visual field loss, and decreased visual acuity. Further investigation revealed the presence of anti-retinal antibodies in serological testing. Patients were diagnosed, therefore, with autoimmune retinopathy. The authors highlighted that, overall, 15 cases of retinopathy attributed to immunotherapy can be found in the literature. Those cases indeed are rare, yet they shed light on the potential need for screening patients before ipilimumab or other ICIs [13]. Additionally, a peculiar case involving a patient with confirmed lung cancer received durvelumab+tremelimumab, developed central retinal vein occlusion, and was managed successfully with intravitreal bevacizumab injection [14].

In a recent systematic review, optic neuritis with acute visual loss and optic disc edema were commonly associated with ipilimumab compared to other immunotherapy agents. Also, neuroretinitis with manifestations of eye pain, redness, photophobia, and metamorphopsia was seen in one patient who received ipilimumab. The combination of both ipilimumab and nivolomab was correlated with a higher incidence of bilateral myasthenia gravis with diplopia, ptosis, and ophthalmoplegia. Corresponding to the autoimmunity that occurred, patients may also develop what is known as thyroid-like eye disease (TED). The resultant clinical features of this realm include proptosis, diplopia, periorbital edema, conjunctival injection, and chemosis. Fortunately, the clinical course of these neuro-ophthalmic complications generally improved with high-dose corticosteroid therapy or immunosuppressants with discontinuation of immunotherapy. This systematic review sheds light on the importance of prompt recognition and management of such adverse events [15].

In advanced malignancies, ICIs have been shown to enhance prognosis. Depending on the ligand that they are targeting, checkpoint inhibitors come in several different types. PD-1 inhibitors such as pembrolizumab and nivolumab are well-known examples. However, they may cause extensive ocular irAEs. Even though they are quite rare, they can happen, accounting for about 1% of treated patients, especially since their significance is growing as more indications and immunotherapy are needed. Ocular adverse events can vary from transient blurriness of vision to total vision loss. Leading to serious impairment of the patient's quality of life and posing a threat to their vision [16]. The exact causes of why these ocular irAEs are happening are not fully understood. However, it is now known that blocking checkpoints activates T-cells, which can target both tumor and healthy cells. An auto-immune reaction results from this, which can affect any organ, including the eyes. It is believed that ICIs directly cause immune-related adverse effects by stimulating an off-target cellular immunological response [17]. A theory of how PD-1 inhibitors can affect the eye is that retinal pigment epithelium cells in the eye constitutively express PD-1. When blocked, it can cause an immunological response against intraocular tissues [16]. 

Different areas of the eyes were shown to be affected by PD-1 inhibitors. Orbital adverse events were reported, such as myositis secondary to pembrolizumab use, with inflammatory infiltrates seen on the biopsy. Ophthalmoplegia and ptosis were reported as the presenting features. In addition, thyroid-like orbital inflammation has been reported, which was eventually resolved with steroids. Regarding the cornea, dry eye is among the most common adverse events. Moreover, Sjogren-like syndrome has been established with the use of PD1/PD-L1 inhibitors. Along with anterior uveitis being one of the most common side effects. Also, VKH-like uveitis has also been reported with the use of nivolumab [18]. The hypothesis around that is that an immune response against melanoma also attacks normal melanin-containing tissue, which results in VKH illness [17].

A case report demonstrated a patient with bilateral anterior chamber reaction and choroidal, which resolved after giving the patient both intraocular and systemic steroids. As well as that, there have been reports of pembrolizumab causing Bechet's syndrome. The patient experienced corneal erosions as ocular symptoms and developed oral and genital ulcers as systemic manifestations of the syndrome. Apart from this, Nivolumab has been reported to induce antiretinal antibodies, causing MAR-like retinopathy. Optical imaging showed hyperfluorescence with damage to the function of the rod/cone cells, as observed on the electroretinogram. In addition to this, in a single case, a patient experienced choroidal detachment while taking ipilimumab/pembrolizumab combination therapy. Neuropathy was also reported in some cases. Pembrolizumab has been associated with the development of ocular myasthenia, characterized by the production of anti-acetylcholine receptor antibodies. Also, cases of optic neuritis have been documented in association with pembrolizumab. Early recognition of the condition allowed early treatment with steroids, resulting in the preservation of vision. Furthermore, nerve palsies have been reported with pembrolizumab (abducens), which resolved after drug cessation and giving the patient high-dose steroids [18].

A retrospective cohort study was conducted at a tertiary cancer center, examining a total of 1280 patients who received ICIs between 2010 and 2020. One hundred and thirty patients were found to have ocular toxicity. These patients represented 10% of the study population. Patients may experience more than one irAE, such as dry eye and retinopathy. Sixty-one years was the mean age of the patient population (age range, 25-88). Among the patients identified, the rates are highest in white individuals, followed by Hispanics, and lowest in blacks, African Americans, and Asians. In addition, rates are slightly higher in males compared with females. Moreover, the mean time to develop ocular toxicity related to an ICI was 6.1 months, based on the first dose of the ICI administered. The minimum time was six days, and the maximum was 36 months. In the study, the most commonly treated malignancy with ICIs that resulted in ocular irAEs was metastatic melanoma. This was followed by renal cell carcinoma and lung cancer. After the results, it was determined that ocular irAEs were most commonly associated with PD-1 inhibitors, with nivolumab being the most frequently reported, followed by pembrolizumab. Adding to that, corneal toxicity was the most common ocular irAE, with dry eyes being the most frequently encountered specific ocular irAE. This was followed by neuro-ophthalmic disorders, hence optic nerve pathology being the most common. Other common toxicities were retinopathy, uveitis, scleritis, and periocular complications [16]. Another retrospective registry study was designed to determine the incidence and recurrence rates of ophthalmic immune-related adverse events. It showed ophthalmic immune-related AEs are elevated compared with ocular complication rates in the entire population. In addition, patients with a history of prior autoimmune ocular disease are at high risk of recurrence of ocular complications [10].

As regards PD-L1, which is a protein expressed in ocular tissues and is thought to play a role in antitumor activity, it is also implicated in the prevention of autoimmune reactions against the eye [19]. Ocular complications are possible adverse events related to PD-L1 inhibitors. It is estimated that the prevalence of moderate-severe ocular complications of PD-(L)1 inhibiting agents is 0.4%, with an incidence of 0.7 per 1000 individuals per month, as determined by a prospective cohort study. Ophthalmic events related to PD-(L)1 inhibiting agents are divided into intraocular events, ocular surface events, and ocular neuromuscular events. Intraocular events ranged from anterior uveitis to panuveitis, which may have unspecific features, including retinal vasculitis, vitritis, papillitis, retinal serous detachment, chorio-retinal lesions, or uveal effusion. This can be controlled by topical steroids. Dry eye disease is usually the most common form of ocular surface events and is usually the first and most common event reported by patients using ICIs (it was previously reported to have a prevalence of 1%). Other surface diseases include episcleritis and blepharitis. Dry eye disease might also be induced as a part of a systemic syndrome produced by anti-PD-(L)1 agents, like Sjogren syndrome or sarcoidosis. Neuromuscular ocular symptoms may include orbital myositis and ocular myasthenia gravis. Orbital myositis has been associated with concomitant myocarditis [20].

One review, which reviewed the most commonly used agents, reported that the most commonly reported agent even related to atezolizumab was uveitis (1%). As for Durvalumab, keratitis and uveitis were reported (1% each), along with other events like ocular myasthenia, extraocular myopathy, and non-specified eye involvement when combined with Tremelimumab [21]. In another study that investigated the association of ocular events with immunotherapy, it was found that the anti-PD-L1 inhibiting agent atezolizumab was the most common agent related to the eye inflammatory process. While the other PD-L1 inhibitor, Durvalumab, was not significantly associated with ocular events [22]. Furthermore, data from a retrospective analysis of results from two adverse events databases showed that the agent atezolizumab was the agent causing routine ocular events in 20 patients from the total number of patients with eye-related adverse events (9%), and none from the Durvalumab group. As for the serious ocular events, six of them (13%) were on atezolizumab (sclerouveitis, paralytic lagophthalmos, bilateral blurry vision, and episcleritis), and only two (4%) of them were on Durvalumab (diplopia) [23].

In another study that investigated 31 patients with ocular symptoms who are on PD-1/PD-L1 inhibitors, it was found that Durvalumab is the agent taken by three of the 31 individuals. In those three patients, each of them had a different event (unilateral cranial nerve involvement, bilateral neuromuscular junction disturbance, and unilateral muscle/orbit event). The patient with muscle/orbit disorder had presented with diplopia. He was found to have immune-mediated myositis on biopsy, with an outcome of persistent diplopia and exotropia of the left eye. As for the patient with a cranial nerve disorder, he initially presented with ptosis and diplopia and was later found to have third cranial nerve palsy. He was stable on treatment with prednisolone. The same presentation of diplopia and ptosis was found in the patient with neuromuscular junction disease, but it was found later on to have a positive titer of anti-titin antibodies (but negative anti-acetylcholine antibodies) and positive electromyography for myositis. Resolution of all symptoms was achieved for this patient by discontinuation of Durvalumab and by starting him on pyridostigmine, prednisolone, intravenous immunoglobulin (IVIG), and mycophenolate. The median time for the onset of symptoms among all 31 individuals was less than 3.5 months [24].

Factors related to the development of ophthalmic complications were investigated in a study conducted on 40 patients. It was found that the use of atezolizumab was associated with the development of foveal interdigitation zone thickening/serous retinal detachment (IZT/SRD) using univariate logistic regression. On multivariate regression, ocular trauma/surgery and the use of Pembrolizumab were associated with the development of intraocular inflammation, while the use of BRAF/MEK inhibiting agents and brain metastasis were associated with IZT/SRD and neuro-ophthalmic complications, respectively [14]. Also, atezolizumab was implicated in multiple case series and case reports for the development of different kinds of eye disease (uveal effusion, uveitis, retinal detachment, and optic nerve damage), while Durvalumab was mainly associated in these case reports and series with development of orbital myositis [19].

Another study from the Mayo Clinic, which retrospectively reviewed the files of 996 patients who are taking ICIs, showed that patients who are using PD-L1 inhibitors had older ages at presentation as compared to those using PD-1 inhibitors, CTLA-4 inhibitors, and combination therapy. PD-L1 patients, when compared to the other groups, also had a higher frequency of ocular surface side effects (7%, as compared to 0% for the CTLA-4 group, 2% for the PD-1 group, and 3% for the combination group) [25]. In another retrospective analysis, uveitis was significantly associated with PD-1/PD-L1 inhibiting agents in all cancer types. Also, uveitis occurs more commonly in melanoma patients as compared to lung cancer and renal cell carcinoma. Retinal detachment was also associated more with melanoma, while optic nerve disorders and lacrimal gland disorders had comparable results in all cancer types. Combining anti-PD1 and anti-PD-L1 agents with an anti-CTLA-4 agent increased the chances of developing ocular events [26].

Ototoxic events related to immune checkpoint inhibitors

CLTA-4 inhibitors, which work by boosting the immune response against tumors via blocking CTLA-4, have a negative effect on the immune system when they have a high level of expression. It has a favorable effect on oncological patients. However, many side effects were reported; mainly the skin and the alimentary tract were affected [27]. Cochleovestibular side effects are few and far between [28].

Two cases of a case series showed ototoxic events related to ipilimumab and nivolumab. The first case had bilateral moderately severe high-frequency sensorineural hearing loss, decreased word recognition in both ears (right 88%; left 92%), which was detected on audiogram examination, tinnitus, and clogging sensation of the ear, which occurred three weeks after developing other side effects of ICI. The second case developed tinnitus, dizziness, and hearing loss, which were progressive for three months. An audiogram was performed for this case, which showed moderate to severe bilateral sensorineural hearing loss (SNHL) and decreased speech recognition in the left ear but preserved word recognition (right: 96%, threshold: 35 dbl; left: 100%, threshold: 50 dbl). It’s worth mentioning that these two cases were diagnosed with ototoxicity secondary to ICI because other factors causing vestibulocochlear events were excluded [5]. Also, a recent case report highlights vestibulocochlear events in a patient treated for cutaneous melanoma. Following ipilimumab and nivolumab therapy, the patient developed sudden bilateral hearing loss alongside symptoms such as dizziness, imbalance, and tinnitus. Despite initial improvement with steroid treatment, the patient experienced recurrent episodes of hearing loss, necessitating additional interventions, including intratympanic dexamethasone injections and infliximab therapy. Subsequent audiograms demonstrated significant improvement and stability of hearing, emphasizing the impact of vestibulocochlear adverse events in ipilimumab and nivolumab therapy [29]. 

A noteworthy case series highlighting a patient who had stage IV melanoma and received a combination of ipilimumab and nivolumab developed positional nystagmus, balance disorders, bilateral hearing loss, and brief rotatory vertigo after two months of commencement of ICI. Brain MRI and lumbar puncture showed no abnormalities. Several cochleovestibular tests were performed, which revealed bilateral perceptive hearing loss with slight tonal predominance in the right ear, but speech recognition was symmetrical. The patient had grade 3 hepatitis, which prompted the need to discontinue ICI. The cochleovestibular symptoms resolved after three months of discontinuation of ICI [28]. Therefore, it points out the interpretation of the resolution of vestibulocochlear complications exclusively to the cessation of ICI. Furthermore, a case study of a patient with stage IV metastatic melanoma of the perianal skin who was given ipilimumab and nivolumab encountered an abrupt onset of vertigo and bilateral hearing loss. An evaluation of the case was conducted, including an MRI and lumbar puncture, to exclude potential causes for these symptoms. The diagnosis of ICI-associated aseptic meningoencephalitis was made. Therefore, the patient underwent systematic steroids and intratympanic steroids, which resulted in improved hearing. Nevertheless, it was temporary, and the patient developed permanent bilateral profound SNHL [30]. In this case, the development of sudden vertigo and bilateral hearing loss after initiation of ipilimumab and nivolumab raises concerns about possible ototoxicity associated with these medications.

As for PD-1 inhibitors, it has been associated with the development of otovestibular dysfunction, manifesting primarily as hearing loss, tinnitus, or difficulty with balance. The mechanism, despite being poorly understood, is thought to be due to the high anti-melanocyte activity of adoptive cell immunotherapeutic agents. Making it highly effective against melanomas, but also reacting against melanocytes elsewhere in the body, including melanocytes of the inner ear, which lie in the stria vascularis (called intermediate cells). Disruption of the intermediate cells results in disturbed maintenance of the potassium-rich endolymph of the scala media, which causes audiovestibular dysfunction [22,31]. A case series of four patients who received ICIs, all of which had a PD-1 inhibiting agent as a part of the regimen (usually Nivolumanb combined with the CTLA-4 inhibiting agent Ipilimumab), showed that ototoxic manifestations included tinnitus (most commonly among the four patients), aural fullness, vertigo, and imbalance. These usually presented with other ICI-related events like uveitis, myositis, headache, and optic neuritis. The usual presentation of ICI-related events is bilateral sensorineural hearing loss, with cases infrequently reported with unilateral involvement. Findings of the audiogram in ICIs-related ototoxicity will show patterns related to the mechanism of hearing loss. A middle ear effusion will show a conductive pattern of hearing loss, while inner ear causes will show sensorineural hearing loss. This highlights the importance of physical examination, which might reveal fluid behind the tympanic membrane, suggesting a middle ear etiology of the hearing loss [31].

In a retrospective analysis, class-specific ototoxicity was found in patients who received monotherapy with two PD-1 inhibitors (Nivolumab and Pembrolizumab). These agents had a significant association with sensorineural deafness and sudden hearing loss when given alone. When combined, an association with the development of otitis media was only found when nivolumab was combined with the CTLA-4 inhibitor Ipilimumab [32]. As PD-1 inhibitors have an enhanced effect against melanocytes, they are proposed to be more likely to cause sensorineural hearing loss, which is usually accompanied by damage to the melanocytes of the uvea (uveitis) and the melanocytes of the skin (vitiligo) [33]. 

A systematic review investigating the effect of ICIs on cranial nerves proposed a mechanism involving the vestibulocochlear nerve for audiovestibular dysfunction. It investigated a series of patients, all of whom had a PD-1 inhibitor agent as a part of their treatment regimen, whether as monotherapy or along with another agent. It revealed that the vestibulocochlear nerve was the second most common nerve to be affected adversely (after the facial nerve), with most patients presenting with hearing loss, vertigo, tinnitus, and vomiting. These were confirmed by bilateral symptoms present with bilateral vestibulocochlear nerve enhancement [34]. Moreover, a case series of six patients with ototoxic manifestations after receiving ICIs (all of whom received a PD-1 inhibitor as a part of their regimen) showed that most of those encountering ototoxicity had other organ involvement, including uveitis, dermatitis, arthritis, pancreatitis, thyroiditis, and even hypophysitis. Five of these six cases had bilateral involvement, and only one had unilateral ototoxicity. The number of doses received before ototoxicity ranged variably among these cases (1, 2, 1, 9, 5, and 12 doses). The severity of SNHL also ranged from mild to profound auditory dysfunction. Interestingly, one of those had been treated with prednisolone and had an objective improvement in hearing, while the others were treated with hearing aids and had a stable objective hearing assessment. A reduction in speech perception and word recognition was also observed in some of them [5]. 

In order to prevent immune evasion and stimulate the body's anti-tumor immunological responses, these medications target immune checkpoint pathways. Cancer cells can use immunological checkpoints, which may have been developed to stop autoimmune reactions, to reduce the immune system's reaction to malignant cells. Immune checkpoints are proteins that help the immune system recognize itself and moderate an overreaction when it might become detrimental. Treatments intended to block these checkpoints, or ICIs, enhance the immune responses specific to malignancies and promote the infiltration of T cells into tumors [35,36].

However, PD-1/PD-L1 inhibitors have side effects that range from mild to severe and life-threatening. Focusing on irAEs, which are a set of symptoms that mimic an autoimmune response, including ototoxicity and other organ inflammatory effects, these will appear after about three months from starting the therapy. Even though anti-PD1/PD-L1 inhibitors are less toxic than anti-CTLA-4, they should not be overlooked by physicians [37,38]. Also, a systematic review showed a total of 38 patients who were using ICI for melanoma had hearing impairment during the therapy. In further investigation, this impairment was the result of bilateral sensorineural hearing loss in more than half of the patients (68.6%). Fortunately, this hearing loss was reversible [39]. A case-control study on rats was done, with two groups, one as the control and the other as the study group, both having six rats (12 in total). The control group received saline, while the other group received two doses of nivolumab (a PD-1 inhibitor). The result of the study showed morphological change between the two groups in their organs, the corti. Some of the subjects in the study group showed degenerative changes in the organ of corti, which supports the possibility that PD-1/PD-L1 inhibitors have ototoxic effects on patients [40].

Despite the advancement in understanding the adverse events associated with immune checkpoint inhibiting agents, notable gaps are still present in the literature, as otologic toxicity is relatively under-recognized. Furthermore, the lack of large-scale, high-quality studies contributes to an incomplete understanding of the toxicities related to immune checkpoint inhibitors. Present data in the literature often focus on certain immune checkpoint inhibitors or a small number of patients, which may underestimate the real effect of ICIs.

## Conclusions

In this study, we concluded that ICIs-related ophthalmic IrAEs are potentially influenced by many factors, including race, class, agent used, age, and medical history. The most commonly reported ocular IrAEs are dry eye, uveitis, ocular myasthenia, retinal disease, and cranial nerve disorders, which are usually bilateral. Ototoxicity, on the other hand, has been underacknowledged in the literature. Nevertheless, ototoxicity manifesting bilaterally with hearing loss, tinnitus, aural fullness, and dizziness has been reported. Other IrAEs were reported to occur commonly with ototoxicity. Ipilimumab, pembrolizumab, nivolumab, and atezolizumab are the agents mostly associated with ICI-related ophthalmic and otologic toxicity, especially when combined. Other patient-related factors that may contribute to ICI-related toxicity include a previous history of ocular immune disorders and the concurrent use of other anti-neoplastic agents. Despite the fact that ophthalmic toxicity is fairly investigated in the literature, data on otologic ICI-related IrAEs remain deficient, and further research into it is advised.
